# Neuraxial analgesia during labor and postpartum depression: Systematic review and meta-analysis

**DOI:** 10.1097/MD.0000000000033039

**Published:** 2023-02-22

**Authors:** Bin Li, Xiaohui Tang, Tingting Wang

**Affiliations:** a Department of Anesthesia, Changning Maternity and Infant Health Hospital, Shanghai, China.

**Keywords:** analgesia, depression, epidural, labor, meta-analysis, obstetric, postpartum, systematic review

## Abstract

**Background:**

Severe pain has been linked to depression, which raises the question of whether neuraxial analgesia during childbirth is associated with a reduced risk of postpartum depression. This association has been explored, but previous studies did not control or analyze relevant confounders. We conducted a systematic review and meta-analysis to determine the association between neuraxial analgesia and postpartum depression.

**Methods:**

A systematic review was conducted using PubMed, Embase, and the Cochrane Central Register of Controlled Trials. Studies that tested the effect of neuraxial analgesia during labor on depression or depressive symptoms in the first year postpartum were included. Relevant articles were extracted independently by 2 authors.

**Results:**

In total, 14 studies (86,231 women) were included. The association between neuraxial analgesia and the long-term incidence of postpartum depression after childbirth was the risk ratio = 0.75, 95% confidence interval (CI): 0.56–1.00, *P* = .05; *I*^2^ = 79%, *P* < .00001. There was a significant association (pooled risk ratio = 0.55, 95% CI: 0.34–0.90, *P* = .02; *I*^2^ = 55%, *P* = .06) between neuraxial analgesia and the incidence of postpartum depression in the first week after delivery. The subgroup analysis showed a trend suggesting that in Asian populations, those who received neuraxial analgesia had lower postpartum depression rates than those who received non-neuraxial analgesia (risk ratio = 0.57, 95% CI: 0.38–0.86; *P* = .008; *I*^2^ = 82%) at ≥4 weeks after delivery.

**Conclusion:**

Neuraxial analgesia may be beneficial for the short-term and long-term mental effects of parturient women, especially for short term after delivery. High-quality studies addressing the role of neuraxial analgesia during labor and its impact on postpartum depression remain necessary.

## 1. Introduction

Childbirth is one of the most painful experiences a woman will endure and is associated with an increased risk of first-time episodes of psychiatric disorders.^[1]^ The demands of pregnancy and childbirth render patients vulnerable to psychiatric disorders.^[2]^ Women with postpartum psychiatric disorders have high mortality rates, with the highest risk in the first year after diagnosis.^[3,4]^ The most common postpartum psychiatric disorder is postpartum depression (PPD), which is defined by the diagnostic and statistical manual of mental disorders, fifth edition as depression (e.g., sadness, loss of interest, insomnia, and impaired concentration) that occurs any time during pregnancy or within the first 4 weeks after delivery.^[5]^ PPD can lead to complications, such as emotional lability in the mother and pervasive emotional, cognitive, and behavioral effects on the child.^[6]^ Identifying those at the greatest risk of developing PPD has implications for prevent.

The development of PPD is multifactorial and encompasses risks, such as low levels of social support, breastfeeding problems, and neonatal intensive care admission.^[7]^ Labor pain has been correlated with postpartum blues,^[8]^ and the severity of acute postpartum pain has been cited as an independent risk factor for the development of persistent pain and depression.^[7]^ Therefore, numerous studies have already explored the association between the use of neuraxial analgesia (NA) to relieve labor pain and the development of PPD.

Interestingly, 2 meta-analyses comparing the effects of epidural and nonepidural analgesia on PPD were published almost simultaneously.^[9,10]^ The results were consistent as the use of epidural analgesia to relieve pain during labor did not seem to affect the likelihood of PPD. However, notably, the heterogeneity of the primary results of the 2 studies was relatively high (37% and 74%, respectively). Moreover, the authors of these 2 studies did not attempt to analyze the causes of such high heterogeneity through sensitivity analysis or stratified analysis. In addition, the 2 meta-analyses collected only the incidence of depression from 4 weeks to 1 year postpartum but did not analyze the incidence of immediate PPD; thus, whether the association between epidural anesthesia and PPD is due to the relief of acute pain or other reasons is still unclear. Third, the original intention of these 2 studies were to explore the effect of effective analgesia on PPD. Effective analgesia during childbirth includes epidural anesthesia and spinal anesthesia, but these 2 meta-analyses only included epidural anesthesia, leading to a result that may not be comprehensive. Fourth, the deadline for the retrieval of the above 2 studies was July 2019, and some high-quality clinical studies were published later, which may affect the results. Considering the above issues, the main purpose of this study is to explore the effect of effective labor analgesia on PPD with the aim to conduct an in-depth evaluation of the latest relationship between the use of NA during labor and the risk of PPD.

We present the following article in accordance with the preferred reporting items for systematic reviews and meta-analyses reporting checklist. The protocol was registered on PROSPERO (CRD42019128767).

## 2. Methods

### 2.1. Information sources and searches

Relevant studies were identified by searching PubMed, EMBASE, and the Cochrane Central Register of Controlled Trials (CENTRAL) on April 15, 2019 without language restrictions, and the search was updated on November 7, 2021. The databases were searched in [All Fields] (PubMed), [All fields] (EMBASE), [All Text] (CENTRAL) by applying the following combination of key terms: (maternal OR mothers OR pregnant women OR delivery women OR puerperal* OR primipara OR nulliparas OR maternity OR parturient* OR expect* mother OR gravida OR gestational woman OR gestational women) AND (epidural OR neuraxial OR spinal epidural OR intrathecal OR local anesthesia) AND (labor OR deliver* OR childbirth OR birth OR labor) AND (depression OR depressive OR blue OR distress OR bipolar OR mood disorder OR anxiety OR stress OR Edinburgh Postnatal Depression Scale scores [EPDS]). The reference lists of studies that met the search criteria were also examined to identify additional studies, as well as forward citations and relevant review articles were reviewed. After obtaining all related records, we verified the results and removed duplicates. Then, the titles and abstracts were screened to remove those that were evidently irrelevant.

### 2.2. Inclusion and exclusion criteria

The inclusion criteria were as follows: Observational studies (case-control, cohort and cross-sectional studies); Studies comparing neuraxial and non-neuraxial analgesia in PPD; and; Women who planned spontaneous vaginal deliveries. The exclusion criteria were as follows: Preclinical studies, review articles, meeting abstracts, case reports, case series, editorials, correspondences, and nonhuman studies and; Studies failing to evaluate PPD outcomes after delivery or unspecified timing of the assessment. Non-neuraxial analgesia (Non-NA) included systemic opioids, nitrous oxide, nonpharmacological approaches or no analgesia. Data were extracted from studies providing the presence of NA, EPDS, Beck Depression Inventory, diagnostic, and statistical manual of mental disorders diagnosis of postpartum depression, or diagnosis according to the judgment of a health professional (e.g., GP, health visitor or psychiatrist).

### 2.3. Study selection and data extraction

The data were retrieved independently by 2 researchers (B.L. and X.T.); disagreements were considered by a third researcher (T.W.) and discussed until a consensus was reached. One researcher (T.W.) designed a standard data extraction form in Excel, and the other researchers (B.L. and X.T.) amended and validated the design of this form before it was used for the data extraction. The authors of the studies were contacted (by T.W.) and asked to provide missing data when possible. Information regarding the general characteristics of the study (first author, country and year of study), participants (characteristics of the population, parity and number), experimental interventions (study design, methods of analgesia, definition of PPD and assessment time), and outcomes were extracted.

Notably, although the diagnostic and statistical manual of mental disorders-5 (diagnostic and statistical manual of mental disorders-5) criteria present onset of mood symptoms during pregnancy or within 4 weeks following delivery, the greatest incidence of new depression postpartum occurs 2 to 3 months after parturition.^[11]^ In addition, growing evidence suggests that high antepartum or peripartum depression is a strong predictor of PPD.^[12–15]^ Accordingly, the primary outcome of this study was the incidence of long-term PPD after delivery (4 weeks after delivery). The secondary outcome was the incidence of short-term PPD after delivery (within 1 week of delivery).

### 2.4. Methodological quality appraisal

The risk of bias assessment was performed using the risk of bias in nonrandomized studies-of interventions (ROBINS-I) tool for observational studies.^[16]^ The ROBINS-I tool assesses bias in the following 6 domains: confounding, participant selection, intervention classification, departure from intended interventions, missing data, measurement of outcomes, and selection of reported results. For each domain, an outcome of low, moderate, serious, critical or no information regarding risk of bias was recorded. The overall risk of bias judgment was determined through a combination of the 6 domains. The quality of the enrolled trials was evaluated independently by 2 researchers (B.L. and X.T.), and differences in opinion were resolved by discussion with another researcher (T.W.).

### 2.5. Statistical analysis

Review Manager (RevMan version 5.2.5; The Nordic Cochrane Centre, The Cochrane Collaboration, Copenhagen, Denmark) was utilized for the data analysis. A *P* value < .05 was considered statistically significant. The *I*^2^ statistic was used to assess heterogeneity, and *I*^2^ > 50% was considered indicative of significant heterogeneity. When the *P* value of the heterogeneity test was >.1, multiple similar studies were considered to have homogeneity, and a fixed-effects model was used. A random-effects model was used for the data analysis in cases of heterogeneity (*P* < .1).

The sources of heterogeneity were investigated by analyses of prespecified subgroups defined by the study design (prospective or retrospective), methods of Non-NA (pharmacologic or nonpharmacologic analgesia), parity and racial differences. To control the Type I error rate in multiple hypothesis testing, we used the Bonferroni correction as follows:


$$\begin{array}{l} \alpha *=\frac{\alpha }{c} \end{array}$$

where α* is our new alpha level, α is our a priori significance level of 0.05 for the family of comparisons, and *c* is the number of comparisons.^[17]^ Sensitivity analyses were conducted to assess the robustness of the data by removing each study sequentially. Potential publication bias was assessed with a funnel plot. In the absence of bias, these plots resemble a symmetrical inverted funnel.

## 3. Results

### 3.1. Description of studies

**Figure 1.**
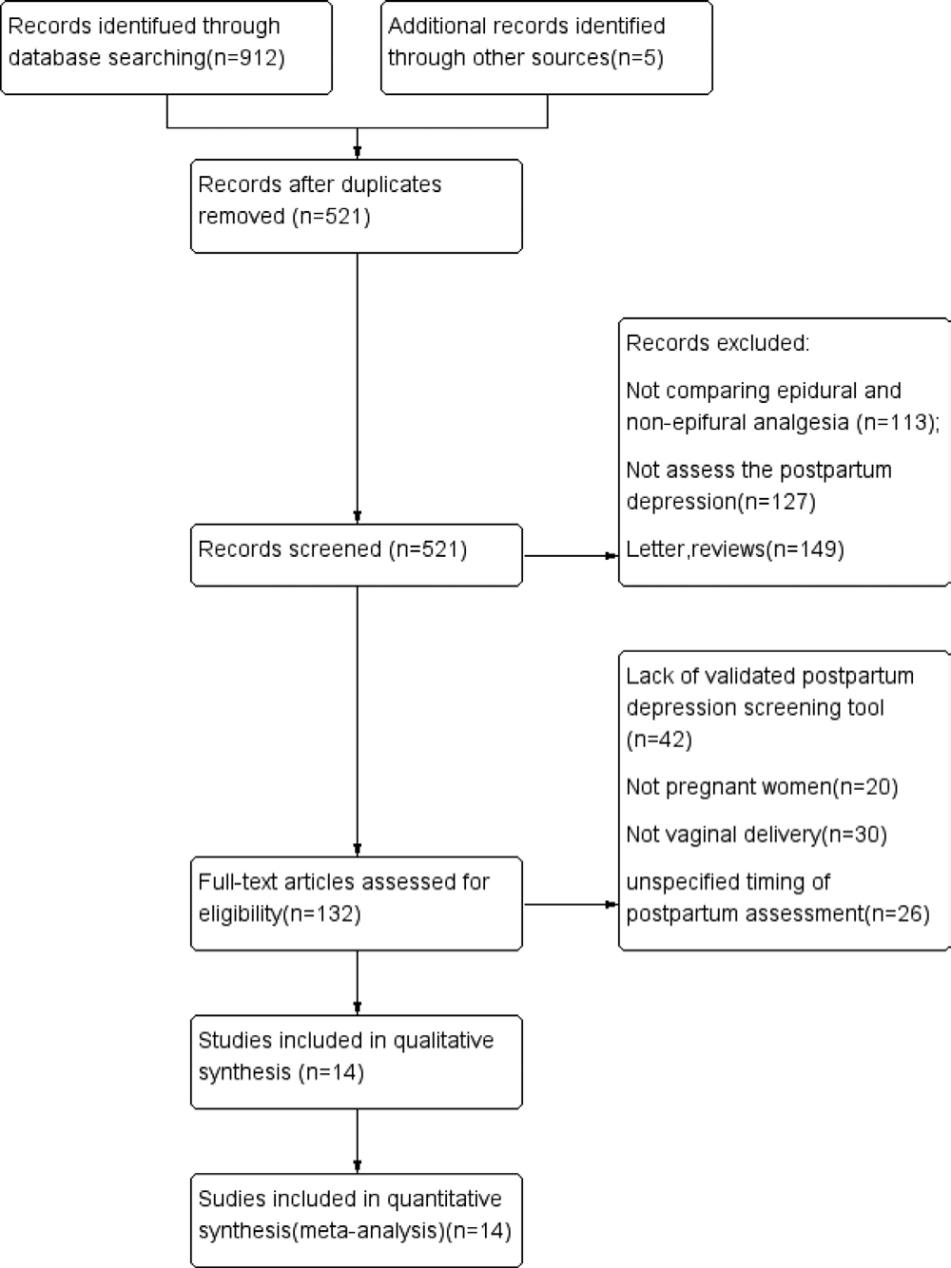
Flow diagram of the search process. EPDS = Edinburgh Postnatal Depression Scale scores.

In total, 912 related studies were obtained from the database search, and an additional 5 articles were found when scanning the article reference lists, yielding a total of 917 initial titles. Five trials required inquiries regarding the data, but only 1 author replied and provided data for 3 days postpartum.^[18]^ Three hundred ninety-six studies were removed because they were duplicates. We excluded another 77 studies after the initial review of the title and 312 studies after the abstracts were reviewed. In total, 132 studies were considered relevant and were read in full. After reviewing the full texts, 14 trials (published between 2004 and 2021) were selected for inclusion (Fig. 1). No unpublished studies in clinicaltrial.gov met the inclusion criteria.

### 3.2. Characteristics of the included studies

The general characteristics of the published articles included in this meta-analysis are shown in Table 1. Thirteen studies involving 86,088 patients were included in the analysis of the association between NA and the incidence of PPD at ≥ 4 weeks after delivery.^[18–28,30,31]^ 43,808 subjects were in the NA group and 42,285 subjects were in the Non-NA group. Five studies involving a total of 1549 patients were included in analysis of the association between NA and the incidence of PPD in the first week after delivery.^[18,20,22,29,31]^ There were 2 retrospective studies^[21,26]^ and 12 prospective studies.^[18–20,22–25,27–31]^ Four studies included nulliparas,^[18,21,24,30]^ 9 studies recruited mixed-parity parturient women^[19,20,22,23,25–29]^ and 1 study recruited multiparas parturient women. ^[31]^Among the included studies, 10 trials involved Non-NA that did not include any drugs or analgesia measures.^[18,19,22–25,27–29,31]^ In 4 other studies, Non-NA referred to the use of general analgesia, narcotics, nitrous oxide, pudendal analgesia, acupuncture, doula assistance or transcutaneous electrical nerve stimulation and meperidine.^[20,21,27,30]^ Seven studies involved Asian populations,^[18,20,24,26,28,30,31]^ and 7 studies involved non-Asian populations. ^[19,21,22–24,28]^

**Table 1:** Characteristics of eligible trials.

Trial	Country/ethnic origin	Period of study	Age	Groups	Sample size (n)	Analgesic methods	Study design	Definition of PPD	Parity	Assessment time	Outcomes
Nahirney 2017 ^[19]^	Canada	2010	15-35	NA	118	NA	PC	EPDS ≥ 10	Mix	Either 6 weeks or 6 months after delivery	No significant difference
				Non-NA	88	Non-NA					
Zhang 2018^20]^	China	Between September 2012 and October 2013.	20–35	NA	213	EP: puncture point:L2-L3 drugs: ropivacaine + sufentanil	PC	EPDS ≥ 10	Mix	Three days and 4 weeks after delivery	No significant difference
				Non-NA	352	Doula and transcutaneous electrical nerve stimulation					
Wu 2018 ^[21]^	Canada	Between April 1, 2006 and March 30, 2012.	18–49 yr	NA	40303	EP or CSE, or combined with other analgesia (general, narcotics, nitrous oxide, nonpharmacological or pudendal)	RC	An outpatient psychiatry visit for a depressive disorder	Nulliparous	Within twelve months after delivery	No significant difference
				Non-NA	40303	General, narcotics, nitrous oxide, nonpharmacological or pudendal					
Ding2014 ^[18]^	China	Between May 1 and August 10, 2009	NR	NA	107	Puncture point:L2-L3 drugs: ropivacaine + sufentanil	PC	EPDS ≥ 10	Nulliparas	Three days and 6 weeks after delivery	PPD was significantly less in NA group
				Non-NA	107	no analgesics					
Riazanova 2018 ^[22]^	Russia	Between December 2015 and March 2017	NR	NA	107	EP: ropivacaine	PC	EPDS ≥ 10	Mix	Three days and 6 weeks after delivery	No significant difference
				Non- NA	103	Not receiving any analgesia					
Orbach-Zinger 2018 ^[23]^	Israel	Between June 2015 and March 2016	≥18yr	NA	932	EP	PC	EPDS ≥ 10	Mix	Six weeks after delivery	No significant difference
				Non-NA	394	Non-EP					
Liu 2019 ^[24]^	China	Between August 1, 2014 and May 29, 2015	18-34yr	NA	368	EP or CSEA: ropivacaine plus sufentanil	PC	EPDS ≥ 10	Nulliparas	Six weeks after delivery	PPD was significantly less in NA group
				Non-NA	140	no analgesia					
Gaillard 2014 ^[25]^	France	Between November 2007 and November 2009	>16yr	NA	216	EP	PC	EPDS ≥ 12	Mix	Between 6 and 8 weeks after delivery	No significant difference
				Non-NA	48	NR					
Suhitharan 2016 ^[26]^	Singapore	Between November 2010 and October 2013	>18yr	NA	329	EP	RC	Diagnostic and Statistical Manual for Mental Disorders criteria by board-certified psychiatrists	Mix	Four–8 weeks after delivery	PPD was significantly less in NA group
				Non-NA	150	Entonox and intramuscular pethidine					
Tobin 2017 ^[27]^	USA	NR	NR	NA	50	EP	PC	EPDS ≥ 10	Mix	Six–eight weeks after delivery	No significant difference
				Non-NA	15	NR					
Tan 2020 ^[28]^	Singapore	Secondary analysis	>18yr	NA	385	EP	PC	EPDS ≥ 10	Mix	Three months after delivery	No significant difference
				Non-NA	266	Non-EP					
Hiltunen 2004 ^[29]^	Finland	Between January 1st, 1996 and March 31st, 1997	19–44	NA	103	EP or paracervical blockade	PC	EPDS ≥ 13	Mix	First postpartum week	PPD was significantly less in NA group
				Non-NA	39	No analgesia, nitrous oxide and acupuncture					
Deng 2021 ^[30]^	China	August 1, 2014 to May 29, 2015	18–34	NA	417	EP or CSEA	PC	EPDS ≥ 10	Nulliparas	Six weeks after delivery	NA was associated with a decreased risk of 6-week PPD
				Non-NA	160	Routine perinatal care including intramuscular meperidine	PC				
Sun 2020^[31]^	China	February 2017 to February 2018	NR	NA	160	EP	PC	EPDS ≥ 10	multiparas	48h and 42 days	PPD was significantly lower in the NA group at 48 hours and 42days.
				Non-NA	263	No analgesia					

CSEA = combined spinal-epidural analgesia, EPDS = Edinburgh Postnatal Depression Scale, EP = epidural, NA = neuraxial anesthesia, NR = not reported, Non-NA = Non-neuraxial analgesia, PC = prospective cohort, PPD = postpartum depression, RC = retrospective cohort.

### 3.3. Methodological quality of the included studies

The results of the quality assessment are presented in Table 2. The ROBINS I tool indicated an overall low to moderate risk of bias, which, in most studies, originated from the selection of the reported results and the presence of possible confounding factors.

**Table 2:** Risk of bias assessment in selected studies by ROBINS-I tool.

Author; Year	Bias due to confounding	Bias in selection of participants into the study	Bias due to missing data	Bias in measurement of outcomes	Bias in selection of the reported result	Overall bias
Nahirney 2017 ^[19]^	Low	Low	Low	Low	Moderate	Low
Zhang 2018 ^[20]^	Low	Low	Low	Low	Moderate	Low
Wu 2018 ^[21]^	Low	Low	Low	Low	Moderate	Low
Hiltunen 2004 ^[29]^	Serious	Low	Low	Low	Moderate	Moderate
Ding 2014 ^[18]^	Moderate	Low	Low	Low	Moderate	Moderate
Riazanova 2018^[22]^	Moderate	Low	Low	Low	Moderate	Moderate
Orbach-Zinger 2018^[23]^	Moderate	Low	Low	Low	Low	Low
Liu 2019 ^[24]^	Low	Low	Low	Low	Low	Low
Gaillard 2014 [26]	Low	Moderate	Low	Low	Moderate	Moderate
Suhitharan 2016 ^[26]^	Moderate	Low	Low	Low	Low	Moderate
Tobin 2017 ^[27]^	Moderate	Low	Low	Low	Low	Low
Tan 2020 ^[28]^	Low	Low	Low	Low	Low	Low
Deng 2021 ^[30]^	Low	Low	Low	Low	Low	Low
Sun 2020^[31]^	Low	Low	Low	Low	Low	Low

All parameters were assessed for their risk by using a scale that classifies them as low, moderate, serious or critical.

ROBINS-I = risk of bias in nonrandomized studies of interventions.

### 3.4. Association between neuraxial labor analgesia and the incidence of long-term PPD after delivery (4 weeks after delivery)

**Figure 2.**
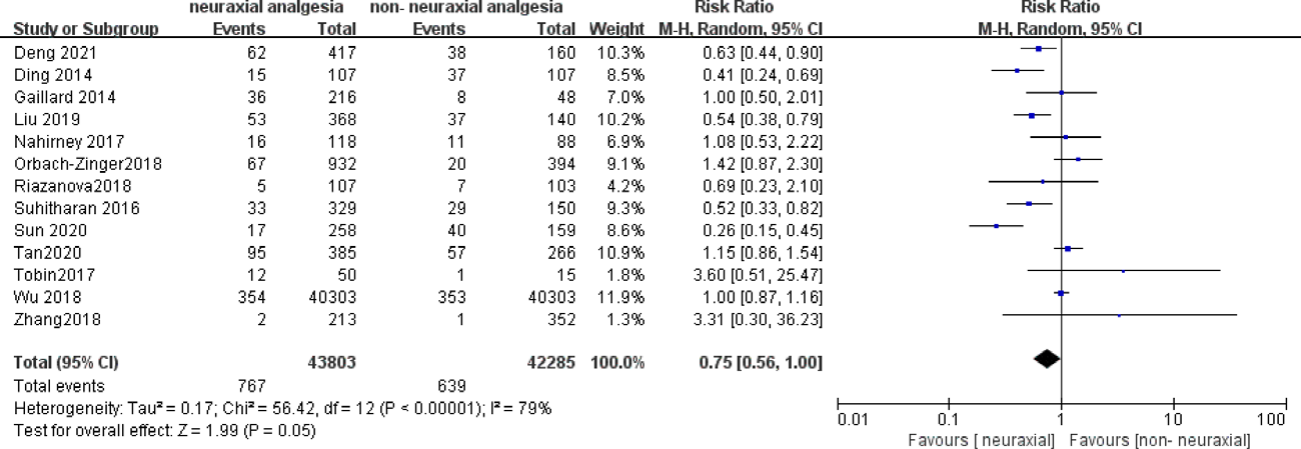
Meta-analysis of studies examining the association between neuraxial labor analgesia and the incidence of PPD ≥ 4 weeks by a random effect model. PPD = postpartum depression, RR = rate ratios.

**Figure 3.**
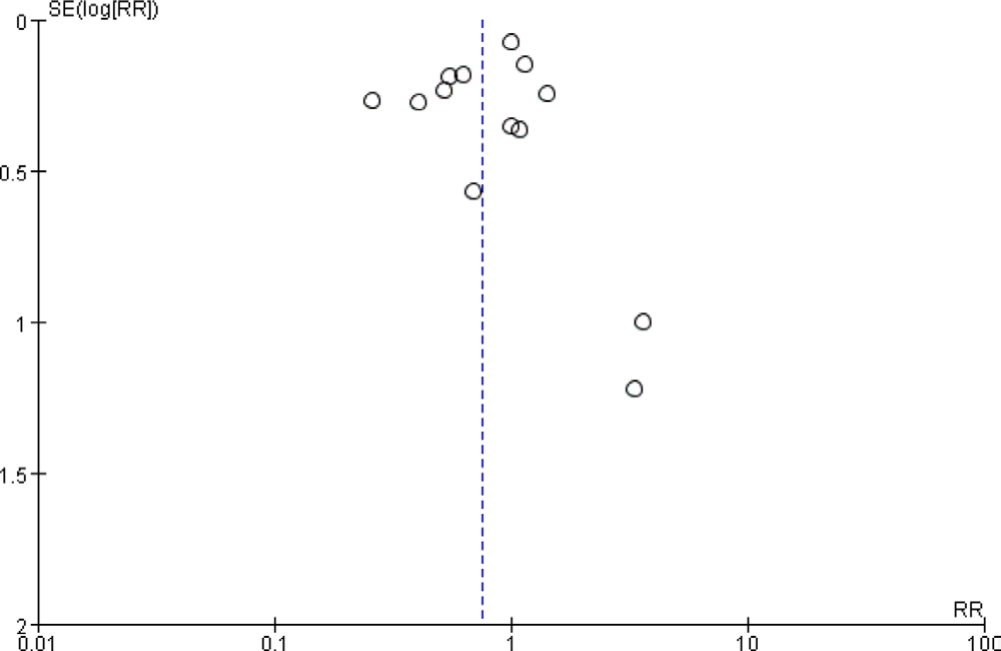
Funnel plot of the incidence of PPD ≥ 4 weeks comparing neuraxial analgesia and non-neuraxial analgesia. PPD = postpartum depression.

Thirteen studies involving 86,088 patients were included in the analysis of the association between NA and the incidence of PPD at ≥ 4 weeks after delivery.^[18–28,30,31]^ The association between NA and the long-term incidence of PPD after childbirth was the risk ratio (RR) = 0.75, 95% confidence interval (CI): 0.56–1.00, *P* = .05; *I*^2^ = 79%, *P* < .00001 (Fig. 2). The CIs were quite wide, and heterogeneity was high. The subgroup analysis showed a trend suggesting that nulliparas who received NA had lower PPD rates than those who received Non-NA (RR = 0.63, 95% CI, 0.42–0.97; *P* = .04; *I*^2^ = 85%). In addition, the subgroup analysis showed a trend suggesting that in Asian populations, those who received NA had lower PPD rates than those who received Non-NA (RR = 0.57, 95% CI, 0.38–0.86; *P* = .008; *I*^2^ = 82%). We sequentially removed each study, and there were no major changes in the direction or magnitude of the statistical findings. No evidence of publication bias was evident by visual inspection of the funnel plot (Fig. 3).

### 3.5. Association between neuraxial labor analgesia and the incidence of short-term PPD after delivery (within 1 week of delivery)

**Figure 4.**
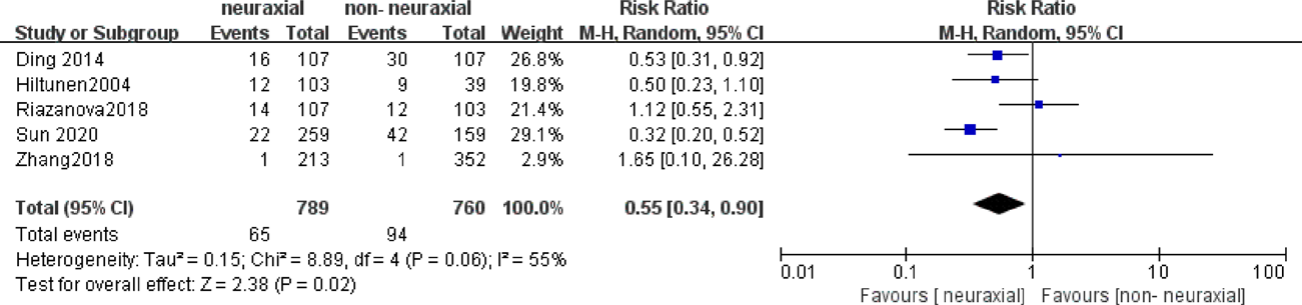
Meta-analysis of studies examining the association between neuraxial labor analgesia and the incidence of PPD at 1 week after delivery by a random effect model. PPD = postpartum depression, RR = rate ratios.

**Figure 5.**
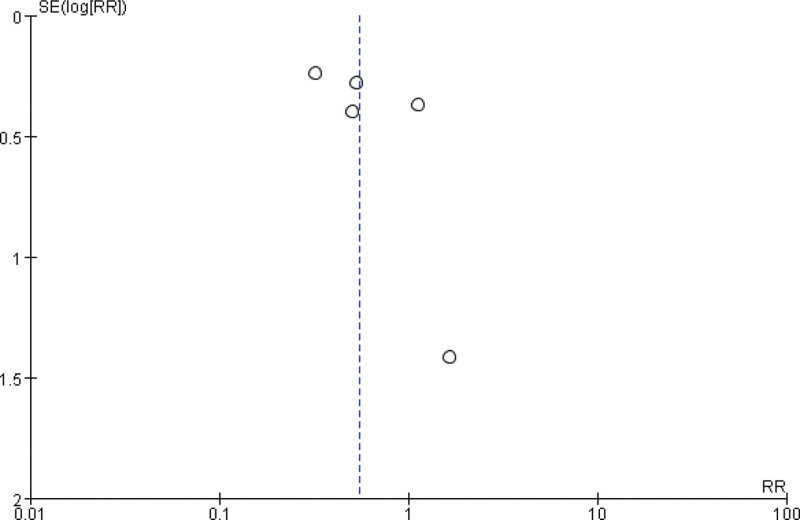
Funnel plot of the incidence of PPD 1 week after delivery comparing neuraxial and non-neuraxial analgesia. PPD = postpartum depression.

Five studies involving a total of 1549 patients were included in the analysis of the association between NA and the incidence of PPD in the first week after delivery.^[22–24,29,31]^ There was a significant association (pooled RR = 0.55, 95% CI: 0.34–0.90, *P* = .02; *I*^2^ = 55%, *P* = .06) between NA and the incidence of PPD in the first week after delivery (Fig. 4). The subgroup analysis showed that NA was a significantly associated with the incidence of PPD in the first week after delivery compared with Non-NA (nonpharmacologic analgesia) (pooled RR = 0.49, 95% CI: 0.36–0.67, *P* < .00001; *I*^2^ = 76%, *P* = .02) (Table 3). We sequentially removed each study from the analysis. When Ding was deleted,^[29]^ the result changed (pooled RR = 0.58, 95% CI: 0.29–1.19, *P* = .14; *I*^2^ = 66%, *P* = .03). No obvious asymmetry was detected in the funnel plots (Fig. 5).

**Table 3:** Subgroup analysis of the outcomes.

	Outcomes									
Study group	Number of studies (n)	Number of participants (n)	Test of association			Test of heterogeneity				
			RR	95%CI	*P* value	Model	*P* value	*I* ^2^	*I*^2^ test for subgroup differences (%)	*p* for the interaction between subgroup and treatment
PPD incidents (≥4 wk after delivery)										
*Study design*										
Prospective	11^[18–20,22–25,27,28,30,31]^	5003	0.76	0.53–1.10	.14	RE	<.00001	78	0	0.96
Retrospective	2^[21,26]^	81085	0.75	0.40–1.41	.37	RE	.008	86		
*Nulliparas*										
Yes	4^[18,21,24,30]^	81905	0.63	0.42–0.97	.04	RE	.0001	85	65.4	0.09
No	8^[19,20,22,23,25–28]^	3766	1.01	0.73–1.41	.94	RE	.05	49		
*Nonpharmacologic analgesia*										
Yes	9^[18,19,22–25,27,28,31]^	3861	0.78	0.50–1.21	.27	RE	<.00001	82	0	0.89
No	4^[20,21,26,30]^	82227	0.75	0.49–1.14	.18	RE	.006	76		
*Asian populations*										
Yes	7^[18,20,24,26,28,30,31]^	3411	0.57	0.38–0.86	.008	RE	<.0001	82	86.2	0.007
No	6^[19,21–23,25,27]^	82677	1.03	0.90–1.18	.57	RE	.0001	70		
PPD incidence (<1 wk after delivery)										
*Nonpharmacologic analgesia*										
Yes	3^[18,22,31]^	842	0.49	0.36–0.67	<.00001	FE	.02	76	0	0.73
No	2^[20,29]^	707	0.57	0.27–1.19	.14	FE	.42	0		

*I*^2^, a test for heterogeneity, *I*^2^ > 50% indicates substantial heterogeneity.

CI = confidence interval, FE = fix-effect model, PPD = postpartum depression, RE = random-effect model, RR = risk ratio.

## 4. Discussion

This meta-analysis showed a trend suggesting that women who received NA had lower PPD rates than those who received Non-NA in long-term (4 weeks after delivery) and short-term (within 1 week of delivery) after delivery. Before firm conclusions can be drawn, caution is advised in interpreting this result given the limited trials and moderate quality of the included studies.

To find the source of the high heterogeneity of the primary outcome (*I*^2^ = 79%), we conducted a subgroup analysis of the Non-NA used, study design, whether nulliparas, and Asian populations were present. The CI was still wide, and heterogeneity was still high. However, interestingly, when the Asian population was excluded, the heterogeneity decreased to 0, suggesting that racial differences had an impact on heterogeneity. This finding is very meaningful, and no such interesting conclusion was drawn in the other 2 meta-analyses.^[9,10]^ Of the 7 included studies involving Asian populations,^[18,20,24,26,28,30,31]^ 5 studies included women from the Chinese population.^[18,20,24,30,31]^ The development rate of labor analgesia in China has rapidly increased over the past decade, but there is still a gap at the international level. The obstetric group of the Chinese Society of Anesthesiology performed a series of surveys investigating labor analgesia across the country, polling 42 maternity hospitals, which included 1,489,228 delivery cases in 3 years. The labor analgesia rates were listed as follows: 11.65% in northeast China, 29.97% in northern China, 30.77% in east China, 17.97% in north China, 11.65% in southern China, 19.66% in central China, 1.02% in northwest China, and 7.56% in southwest China.^[32]^ Due to the low prevalence of childbirth analgesia, women willing to undergo labor analgesia may feel more pain during delivery and, therefore, have a stronger need for labor analgesia in China. Orbach-Zinger et al identified that women’s high willingness to plan labor analgesia and high labor satisfaction have a certain protective effect on PPD.^[23]^ Therefore, the possibility of selection bias is increasing. Although the sources of heterogeneity were identified, the results should be cautiously interpreted because they are based on a limited number of subgroup analyses.

Our secondary purpose was to assess the mental state of women short term after delivery, which can better reflect the association between effective epidural analgesia during delivery and the reduction in PPD symptomatology. Interestingly, the results showed that NA during labor has a protective effect on PPD immediately after delivery (pooled RR = 0.55, 95% CI: 0.34–0.90, *P* = .02; *I*^2^ = 55%, *P* = .06). This finding suggests that short term acute pain improvement during childbirth is related to PPD. However, PPD is affected by various factors, such as a poor marriage status, a poor family economic status, negative life events, lack of social support, initiation of breastfeeding, and social support.^[33]^ The long term mental state after childbirth is affected by the above factors, which weaken the advantage of labor analgesia over time. Therefore, the trend of reducing the incidence of PPD in the long-term after labor analgesia is not so obvious compared with that in the short-term. This result provided meaningful inspiration, that is, effective labor analgesia can benefit the short-term mental state of women after childbirth. However, because of the small sample size of this study, the lower limit of the 95% CI approaching 1 suggests that differences in clinical significance cannot be confirmed with confidence.

Notably, the results of the quality assessment indicated an overall low to moderate risk of bias according to the ROBINS-I tools. ROBINS-I is particularly useful for those undertaking systematic reviews that include nonrandomized studies.^[16]^ In the included literature, 1 article had serious bias due to confounding because the analgesic methods included epidural analgesia or paracervical blockade; thus, confounding factors that affect the outcome of the intervention may exist.^[29]^ Five articles had moderate bias due to confounding bias in the selection of participants into the study or bias in the measurement of the outcomes.^[18,22,25,26,29]^ Therefore, in general, the quality of the included studies was not high, and verification of the conclusion is required.

In the present study, PPD was defined as an EPDS score of 10 or higher or a diagnosis of depression by a psychiatrist after delivery. The EPDS is the most widely used scale to identify PPD.^[34]^ In a recent systematic review by the US Preventive Task Force that used an EPDS cutoff ≥ 10, the sensitivity in identifying major or minor depression in pregnant and postpartum women ranged from 0.63 to 0.84 with a specificity of 0.81, which validated the accuracy of this screening instrument.^[35]^

This meta-analysis had 3 limitations. First, PPD is a multifactorial disorder encompassing multiple medical and psychosocial elements. These elements could not be captured by all of our data sources, leading to an increased risk of confounding bias. Second, as mentioned above, several included studies were of moderate quality and might encompass several systematic biases that could not be solved by this meta-analysis. Third, the observational design cannot exclude unmeasured confounding and selection bias. For example, NA was preferentially offered or requested by women at higher risk of PPD, which, therefore, reduces their risk compared to that of the general population.

In summary, this meta-analysis showed a trend suggesting that nulliparas who received NA had lower PPD rates than those who received Non-NA in long-term (4 weeks after delivery) and short term (within 1 week of delivery) after delivery. Future well-designed prospective studies are needed to confirm our results.

## Author contributions

**Conceptualization:** Tingting Wang.

**Formal analysis:** Tingting Wang.

**Methodology:** Tingting Wang.

**Writing – original draft:** Bin Li.

**Writing – review & editing:** Xiaohui Tang.
